# A Resilient and Effective Task Scheduling Approach for Industrial Human-Robot Collaboration

**DOI:** 10.3390/s22134901

**Published:** 2022-06-29

**Authors:** Andrea Pupa, Wietse Van Dijk, Christiaan Brekelmans, Cristian Secchi

**Affiliations:** 1 The Netherlands Organisation for Applied Scientific Research—TNO, 2316 ZL Leiden, The Netherlands; andrea.pupa@unimore.it (A.P.); christiaan.brekelmans@tno.nl (C.B.); 2 Department of Sciences and Methods of Engineering, University of Modena and Reggio Emilia, 42122 Reggio Emilia, Italy; cristian.secchi@unimore.it

**Keywords:** human-robot collaboration, human-centered robotics, task planning

## Abstract

Effective task scheduling in human-robot collaboration (HRC) scenarios is one of the great challenges of collaborative robotics. The shared workspace inside an industrial setting brings a lot of uncertainties that cannot be foreseen. A prior offline task scheduling strategy is ineffective in dealing with these uncertainties. In this paper, a novel online framework to achieve a resilient and reliable task schedule is presented. The framework can deal with deviations that occur during operation, different operator skills, error by the human or robot, and substitution of actors, while maintaining an efficient schedule by promoting parallel human-robot work. First, the collaborative job and the possible deviations are represented by AND/OR graphs. Subsequently, the proposed architecture chooses the most suitable path to improve the collaboration. If some failures occur, the AND/OR graph is adapted locally, allowing the collaboration to be completed. The framework is validated in an industrial assembly scenario with a Franka Emika Panda collaborative robot.

## 1. Introduction

Industrial applications where human and robots work closely together are becoming the new paradigm of industrial settings [1]. Collaborative robots can take over repetitive, challenging, or dangerous tasks improving the well-being of the operators [2]. There are multiple challenges that emerge from this close collaboration. On one hand, the absence of barriers makes it necessary to pay close attention to how to guarantee the safety of the operator [3,4,5]. On the other hand, it becomes necessary to understand how to create a synergy between humans and robots that is as natural as possible, making the most out of the collaboration [6,7]. Therefore, a strategy on how to allocate and schedule tasks between humans and robots is crucial to improve the human-robot team.

The basis of this strategy is an investigation of how the different tasks that make up the work can be distributed best among the actors in the nominal situation, the task allocation problem. The individual tasks are subject to constraints that prescribe how, when, and by whom the tasks can be executed.

The characteristics of a good task allocation are captured in the fluency concept, which relates to how well the operator and the robot are adapted to each other. Fluent collaboration benefits the work execution and the job-quality of the human operator. These aspects are not captured by optimizing for task efficiency alone. There are subjective and objective metrics for fluency available of which the latter ones can directly be used as an optimization criterion. The objective metrics include the relative portion of functional and non-functional delays of the actors, and the amount of parallel work [8].

The task allocation problem can be solved during the design phase and results in the nominal schedule. This procedure has widely been investigated in other works. Refs. [9,10,11,12] focused on heterogeneous multi-agent task allocation in an industrial setting. While other authors, e.g., [13,14,15], propose to model the human-robot collaboration (HRC) problem as a nonlinear optimization problem. These strategies allow us to find the best nominal schedule.

Even if optimal, a nominal task schedule cannot guarantee a real improvement of the collaboration. In the task execution phase, many factors come into play that cannot be anticipated within the nominal schedule. This requires an efficient and resilient team that can anticipate and adequately respond to these abnormalities. The requirements for an efficient team of human and automated agents, i.e., robots have been formulated in [16] and contributed to a design method in [17]. The requirements and the design method were targeted at general automation challenges and applied in open ended scenarios. The industrial practice is much more constrained and the irregularities that exist have several common causes. Identifying these causes can help the design of resilient solutions for task allocation.

Firstly, it may happen that not all the actors are always available to carry out the collaboration, e.g., the robot has been dispatched to another work station. In this case it would be highly inefficient to again design the job considering only the remaining actors. It would be more convenient to consider this actor availability problem from the beginning and adapt the schedule accordingly. Secondly, the tasks may depend on each other, i.e., there could be precedence constraints, and this interdependence must be considered while ensuring the parallelism between the actors. Thirdly, the operators, as human beings, are inherently different from each other, each with their own skills, capabilities, or individual preferences. Some of them may need or prefer to be guided more during the work, e.g., newly hired workers. For others, on the other hand, an excess of information could be annoying and counterproductive from the job quality perspective. Lastly, there is always the possibility that failures will occur that prevent the correct execution. Therefore, a fallback scenario must be considered when designing a scheduling strategy for HRC scenarios.

This paper firstly presents how an industrial HRC process, with its irregularities during execution time, can be formalized into a set of interdependent tasks. Secondly, it proposes a novel adaptive task scheduling framework for collaborative cells. As a start, the framework uses a database to understand how the collaborative job is composed out of multiple interdependent tasks. Subsequently, at runtime, it monitors the task execution to understand the human operator skills and the task result, i.e., failure or success. This information is exploited to adapt the schedule online, making the framework flexible and able to face most of the situations that may arise in a real HRC scenario.

The main contributions of this paper are:

- A formulation of four primitive situations that encompass most of the scenarios that could occur in a real HRC.
- A novel adaptive task scheduling framework that is effective and applicable to the formalized situations, i.e., suitable for a real industrial application.
- The validation of the proposed framework in several variants, one for each situation, of the same experimental scenario, proving the effectiveness of the framework.

The paper is organized as follows: Section 2 presents the review of related works, while Section 3 formalizes the task scheduling problem for HRC. In Section 4, the overall proposed architecture is detailed, and in Section 5, the scheduling strategy is defined. Section 6 and Section 7 address the handling of different human operators and errors, respectively. Lastly, Section 8 summarizes the experimental validation of the proposed architecture, while Section 9 sums the conclusions and presents suggestions for future research.

## 2. Related Works

Different approaches were presented in the literature to deal with the problem of multi-agent task scheduling. In [18], the author proposes a heuristic method in order to allocate and schedule the tasks between multiple processors. The approach is based on the communication time between the processors and the number of successor tasks, making it suitable for the problem of handling precedence constraints. In [19], a branch-and-bound procedure and a climbing discrepancy search heuristic for the parallel machine scheduling problem with precedence constraints and sequence dependent setup times is proposed. This algorithm can minimize the sum of the completion time and maximum lateness. In [20], the authors propose a Load-Balance Scheduling Algorithm, which allows for allocating and scheduling the tasks in a multiprocessor system. The idea is to use an Earliest Deadline First (EDF) heuristic to first create an *n* ordered tasks list. Then, based on the actual workload, to allocate the task to one processor. In general, these solutions cannot be directly applied in an HRC application, as they consider the presence of homogeneous actors.

In [21], the authors model the HRC working process as a chessboard setting, where the decision of each actor is described by the chess piece move and formulated as a Markov game model. To optimize it, they propose a decentralized Deep-Q-network based MARL (DQN-MARL) algorithm. In [22], the task scheduling is formulated as a Mixed-Integer Linear Programming Problem (MILP) inspired by the Multimode Multiprocessor Task Scheduling Problem. The cost function aims at reducing the total makespan, and the solution is obtained with a constraint programming model and the use of a Genetic Algorithm (GA). In [23], instead, the authors propose the use of a Simulated Annealing (SA) algorithm to find the optimal solution.

These works, however, do not consider the differences between individual operators. As the scheduling procedure adapts to the robot that is available, e.g., considering the robot workspace, it should also be able to modify the schedule based on the human operator that is currently going to perform the collaborative job. In [24], an integrated task allocation and task scheduling strategy for HRC is proposed. The task allocation is solved offline exploiting a two-level feedforward optimization. Furthermore, this strategy is enriched with a feedback procedure based on mutual trust to re-allocate the tasks online. Furthermore, in [25], the authors propose a multi-criteria decision-making framework for task allocation, which generates a solution that best matches the criteria you want to optimize. Moreover, in the case of unexpected events, the algorithm can be exploited for re-scheduling the remaining tasks. In [26], a two-layer dynamic rescheduling framework is presented. The first layer builds the nominal schedule solving offline an MILP problem, while the second layer exploits the real human execution time to reschedule online the tasks. In this paper, the job quality is considered inside the MILP problem both as data to be optimized and as constraints. This work has been further extended in [27] to integrate the scheduling strategy with the safety required by the robot trajectory planner. Moreover, in [28], the authors propose a genetic algorithm that exploits human and robot data, e.g., ergonomics or capabilities, to optimally schedule the tasks in an HRC scenario. The actor characteristics are given offline as input by the user. In [29], a two-level abstraction and allocation for an HRC scenario is presented. The first layer exploits the use of the A* algorithm to optimize a cost function. The second layer, instead, handles the task execution and the respective failures. If the system detects some errors, it is possible to reschedule the tasks recomputing the optimal solution.

Even if they adapt online based on what is currently happening, none of the proposed works are so general to handle all the considered primitive situations at the same time:

1. Scarce resources;
2. Parallelism between the actors;
3. Different human operator skills;
4. Errors during the execution.

The proposed approach focuses on developing a resilient scheduling framework, that is general and applicable to real industrial scenarios. Differently from other works, e.g., ref. [26,30], that are more oriented on the optimization and reduction of the total makespan. The framework does not generate the nominal schedule, it is therefore, to a large extent, supplementary to existing methods.

## 3. Problem Statement

Industrial Human-Robot Collaboration is characterized by multiple agents that work toward a common goal. In this paper, a situation where a human operator *H* and a robot *R* collaborate in a shared workspace, namely the collaborative cell, is considered. The human and the robot have a pre-defined task distribution, which is defined by a set of nominal task schedules. The agents must perform their respective tasks in order to complete the collaborative job. A job typically represents an industrial process, such as the assembly of a product. In this work, it is assumed that the nominal task schedules have already been computed, e.g., exploiting [6,31].

**Figure 1:**
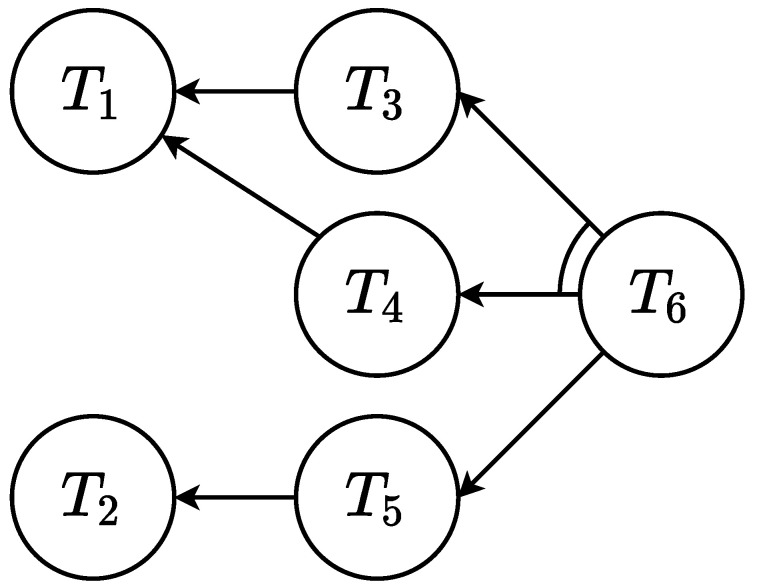
AND/OR graph representation. The unlinked edges represent the OR relations, while the ones connected with an arc represent the AND relations. For example, in the figure, $T_{6}$ can be executed after both $T_{3}$ and $T_{4}$ or after only $T_{5}$.

An effective representation of a general collaborative job can be achieved with an AND/OR graph G=(T,E), as shown in Figure 1 [32]. Each node represents the task, $T_{i}$, while each directed edge $E_{ij}$ means that the parent task $T_{i}$ must be executed after the child task $T_{j}$. Multiple unlinked edges sharing the same parent represent an OR constraint. This constraint imposes that the parent task can only be executed if at least one of the children has been completed, e.g., $T_{4}$ and $T_{5}$ in Figure 1. Thanks to the OR constraint, it is possible to define multiple paths that lead from the same starting point to the same end point, namely *equivalent paths*, e.g., $T_{2}+T_{5}\equiv T_{1}+T_{3}+T_{4}$ in Figure 1. In this work, the first tasks that belong to equivalent paths are defined as *equivalent tasks*, e.g., $T_{1}$ is equivalent to $T_{2}$. When multiple edges share the same parent and are connected with an arc, they model an AND constraint. This constraint imposes that the parent task can be executed only if all the children have been completed, e.g., $T_{3}$ and $T_{4}$. The job is finished when the final task $T_{6}$ is completed; this does not require that all tasks in the job are completed.

Thanks to their structure, the AND/OR graphs intrinsically model both the parallelism between the actors, which is required to reduce the waiting times, and the precedence constraints between the tasks. Furthermore, through the OR constraints it is possible to represent already in the design phase all the different situations that may arise due to the available actor capabilities. This might be the temporary absence of an actor such as a robot that is in maintenance, or an inexperienced operator that needs additional task instructions. In this paper, only human operators are considered to have different skill levels. To handle this, each human task is defined with the minimum expertise level that is required from the operator to be able to perform that task. The robot is assumed to have a fixed set of capabilities that are known during design time, which is common in industrial settings.

Scheduling the task does not ensure that the actor will always perform it correctly. For this reason, each task $T_{i}$ is associated with recovery actions that allow to restore the nominal behavior of the human-robot team. This set of actions can be very complex and, in turn, represented with a AND/OR graph. For ease of reading, this set of actions will be represented in the paper as a single task. Moreover, the collaborative cell is enriched with a task monitoring strategy that allows to check if the task execution has been successful and, in case of failure, it provides information about the error. To achieve this, many algorithms are already available in the literature [33,34,35].

In this work, we aim at designing a scheduling framework that:

- Takes as input the skills of the human operator and its knowledge in the collaborative job, automatically schedules online tasks between the different actors choosing the most suitable path for the situation on the AND/OR tree.
- Automatically handles most situations and the failures that may occur within an industrial scenario, making it suitable for HRC in industrial settings.

## 4. Architecture

**Figure 2:**
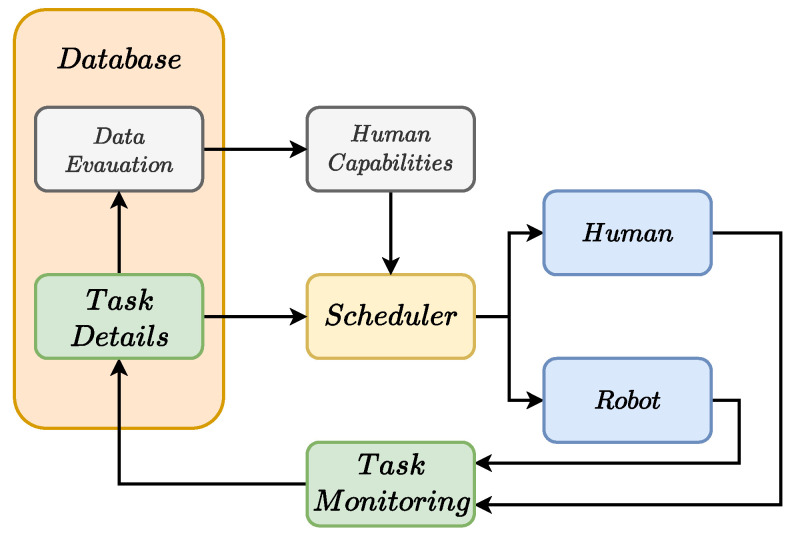
The overall architecture.

The proposed framework is shown in Figure 2, where different components may be distinguished:

- The Database block is responsible for storing all the information regarding the tasks composing the collaborative job, through the Task Details block, and analyzing all the data to evaluate the desired metrics, this is achieved in the Data Evaluation block.
- The Scheduler block, which is the core of the framework, takes care of choosing the most suitable task for each actor online, considering the human operator capabilities and the parallelism in the collaborative job.
- The Task Monitoring block oversees the task execution and communicates with the Database if the task has been completed or not. Moreover, in case of failure, it gives information about the error so that proper recovery actions can be scheduled.
- The Human Capabilities block takes the result of the data analysis to estimate the human operator capabilities and knowledge.

It is worth noting that in Figure 2 the overall framework has been presented. However, the grey blocks are out of the scope of this paper and have only been included for completeness.

The overall procedure starts offline with the design of the AND/OR graph and inserting all the tasks inside the database. Each task $T_{i}$ is associated with its description and requirements, i.e., the actor that must perform it, the precedence constraints as AND/OR constraints, and the minimal required expertise level. For example, $T_{6}$ in Figure 1 can be defined as the following:

$$\begin{array}{cc} T_{6}:\{ & description:{\;}^{\prime \prime }Final^{\prime \prime },\\ \; & requirements:\\ \; & \;\{actor:\;H,\\ \; & \;precedence:{\;}^{\prime \prime }\left(T_{3}\;\wedge \;T_{4}\right)\;\vee \;T_{5}^{\prime \prime },\\ \; & \;level:\;1\}\} \end{array}$$

Subsequently, all these task definitions are passed to the scheduler, which searches along the AND/OR tree to choose, for each actor, the tasks that best suit the current scenario, e.g., operator experience, actor availability. This is achieved, exploiting the data coming from the others blocks. Once a task has been chosen, it is forwarded to the respective actor who must perform it.

At this point, the task monitoring block continuously checks which tasks have been concluded and what the final task result is, i.e., success or failure. This information is also stored inside the database and used for two different purposes. Firstly, in the case of error, it is exploited to choose a proper set of recovery tasks that may be performed in order to continue the collaboration. Secondly, it is used by the data evaluation strategy in order to increase or reduce the human operator expertise level. Finally, the scheduler is triggered again to assign another task, until the collaborative job is concluded.

## 5. Scheduler

The scheduler is the core of the proposed framework and has the goal of distributing the tasks among the available actors, i.e., humans and robots. It requires as input both the AND/OR graph of the collaborative job, already defined in a previous design phase, and the availability of actors along with their skills and capabilities. These two inputs are exploited to choose at runtime the best path for the current scenario, tailoring the specific needs of the human operator during the collaboration. Thus, the scheduler aims at improving the synergy between the actors with a consequent improvement of the HRC.

The entire scheduling pipeline is implemented according to the pseudo-code reported in Algorithm 1.

The scheduler needs as input the AND/OR graph G coming from the database, the set of available actors A, and the actual human operator expertise level $H_{L}$ (Line 1). It immediately sets to false the variable $End_{J}$, which is used to identify when the collaborative job is concluded and instantiate to true the list of Boolean variables $F_{A}$, which indicates if each actor is free in order to start a new task (Lines 2–5).

Then, the algorithm enters a while loop where it continuously executes the overall pipeline until the collaborative job has been finished. Firstly, the scheduler checks for each task *T* if the requirements have been satisfied (Line 9). This is translated in checking whether the respective agent is available to accept a new task and if the precedence tasks have been executed. If this is the case, the algorithm checks if the level of the human operator is sufficient to execute the task (Line 10). This condition allows the scheduler to handle the “Different Operator Skills” situation detailed in Section 6. It is worth noting that if the actor of task *T* is not the human operator, the function checkLevel() always returns true. This is because the robot always has the required skills to perform the tasks assigned to it. If the level check is also successful, the task *T* is scheduled to the respective actor, who is immediately marked as unavailable, and it is added to the list of the tasks to be monitored $T_{A}$ (Lines 11 and 12). At this point, all the equivalent tasks of *T* are discharged and removed from the graph. Since the scheduler works online, this procedure is necessary because otherwise it could happen that the algorithm runs through two parallel paths, which is unnecessary to reach the goal.
**Algorithm 1** Scheduler()1:**Require:**G, A, $H_{L}$2:$End_{J}\leftarrow false$3:**for**a∈A**do**4:    $Free_{a}\leftarrow true$5:    $F_{A}\leftarrow \mathrm{pushback}\left(Free_{a}\right)$6:**end for**7:**while**$End_{J}=false$**do**8:    **for** T∈G **do**9:        **if** $\mathrm{checkRequirements}(T,F_{A})$ **then**10:           **if** $\mathrm{checkLevel}(T,G,H_{L})$ **then**11:               $F_{A}\leftarrow \mathrm{setBusy}\left(T,F_{A}\right)$12:               $T_{A}\leftarrow \mathrm{pushback}\left(T\right)$13:               G←discardEqTasks(T)14:           **end if**15:        **end if**16:    **end for**17:    **for** $T\in T_{A}$ **do**18:        R,E←taskMonitor(T)19:        **if** R=∅ **then**20:           continue21:        **else if** R=Executed **then**22:           **if** isFinal(T)**then**$End_{J}\leftarrow true$23:           **end if**24:        **else if** R=Failed **then**25:           G←applyRecovery(T,E,G)26:        **end if**27:        $T_{A}\leftarrow \mathrm{remove}\left(T,T_{A}\right)$28:        $F_{A}\leftarrow \mathrm{setFree}\left(T,F_{A}\right)$29:    **end for**30:**end while**

Then, the scheduler uses the database to check, for all the active tasks, the information regarding the task monitoring (Line 18). The task monitoring strategy can be implemented using several strategies available in the literature as, e.g., ref. [36,37], and allows to detect critical deviations in the collaboration. In turn, critical deviations from the nominal behavior are translated into a failure, which is stored inside the database. If no results are available, the task is not concluded, and the scheduler continues to inspect the other tasks (Line 20). In the other cases, the behavior of the scheduler depends on the type of the result. If the task has been concluded, the algorithm only checks if this is a final task, and the job is finished (Line 22). If the actor has failed, the scheduler locally adapts the graph in order to generate a recovery procedure (Line 22). This local adaptation depends on the type of error *E* coming from the monitoring and it is further detailed in Section 7. Lastly, the concluded task is removed from $T_{A}$ and the actor is marked as free (Lines 27 and 28).

## 6. Different Operator Skills

By definition, an HRC application is characterized by the presence of both humans and robots. The differences between these two actors are quite intuitive. Human operators are capable of very complex tasks, improving execution every time, but they can hardly reach or work in hazardous environments. Robots, on the other hand, are less affected by hazards in the surrounding environment, but they are not able to intrinsically learn from their last task execution.

Unlike robots, the human is never a constant factor, human operators have different skills, which can be improved or acquired. When starting on a new type of job, it is very likely that a human operator needs detailed instructions on the tasks that need to be performed. Therefore, it becomes useful to divide the work into several tasks that are easy to comprehend, allowing the operator to acquire the necessary skills and knowledge. At some point, the operator will acquire much more experience in the collaborative work, and it may be more convenient to combine some tasks into one, avoiding unnecessary fragmentation. An example can be a complex wiring activity: a new human operator may require wire-by-wire instructions, while for expert operators a single instruction with an overview of all the wires is sufficient.

The framework handles this situation in two different phases: First, during the design of the AND/OR graph and the insertion of the tasks inside the database. Second, adding the check level step inside the scheduler; see Algorithm 1 in Section 5. This constraint is implemented according to the pseudocode in Algorithm 2.
**Algorithm 2** checkLevel()1:**Require:**$T,G,H_{L}$2:a←getActor(T)3:**if**a≠H**then**4:    returntrue5:**else**6:    $T_{E}\leftarrow getEqTasks\left(T,G\right)$7:    **for** $t\in T_{E}$ **do**8:          **if** $t.level>T.level\;\mathrm{and}\;t.level\leq H_{L}$ **then**9:                 returnfalse10:        **end if**11:    **end for**12:    **if** $T.level\leq H_{L}$ **then**13:        returntrue14:    **end if**15:    returnfalse16:**end if**

The level checking needs as input the task to be analyzed *T*, the AND/OR graph G, and the actual human operator expertise level $H_{L}$ (Line 1). It immediately gets the actor *a* that must perform the task and, if it is not a human operator, it returns true allowing to schedule the task (Lines 2–4). If the actor is the human, it is necessary to investigate more to decide if *T* is the most suitable task. The algorithm firstly builds a list of all the equivalent tasks $T_{E}$ analyzing the AND/OR graph (Line 6). Then, for each equivalent task, it checks if there is a task that requires a greater knowledge than the one that is required by the current analyzed task and if this knowledge is still admissible for the current human operator. If this is the case, the algorithm returns false (Line 9). It is worth noting that this part of the code allows to always choose the task that best suits the human knowledge level, improving the collaboration and the job quality for the human operator. If no better tasks are available, the algorithm checks if this task can be performed by the human operator before allowing its execution (Line 13).

## 7. Error Representation

During the collaboration, it is very unlikely that everything happens exactly as planned. Human operators and robots will make errors during the task execution, preventing the nominal behavior. The framework must be able to handle these errors and adapt the behavior of the actors accordingly.

Errors can be classified into two categories based on the way the error is handled:

- *Restorable Error*, which is an error that does not preclude the task and, after some checking and restoring actions, it is possible to retry the execution. This may happen when the robot accidentally hits something and, after the human operator confirms that there are no damages or safety problems, the task is assigned again to the robot, i.e., the repair task brings the product to the state *before* the erroneous task.
- *Non-Restorable Error*, which is an error that precludes the task execution, and it is necessary to execute another task to continue the collaborative job. This may happen when the robot places an object with a wrong orientation and the scheduler assigns a new task to replace the object in the correct pose, i.e., the repair task brings the product to the state *after* the erroneous task.

In the proposed framework, both categories of errors are handled by locally adapting the AND/OR graph. This allows the scheduler to continue working without losing its generality. It is worth noting that some errors are of a type that do not allow the collaboration to continue, e.g., a safety violation. Since these cases require more severe intervention of an operator and a consequent reset of the collaborative job, they are not covered by the proposed framework.

### 7.1. Restorable Error

**Figure 3:**
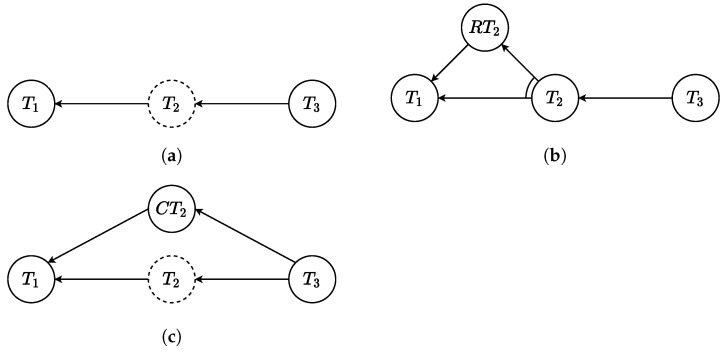
Representation of the errors and the adaptation of the AND/OR graph. The dashed line in (**a**) Means that $T_{2}$ failed. (**b**) Shows the restoring path, while (**c**) shows the corrective one.

After a restorable error occurs, the nominal behavior of the actors could be ideally restored since the execution of the task is not precluded. The way the framework handles this situation is illustrated in Figure 3b.

A new graph path that goes from the previous task to the failed one is generated. This path contains all the tasks that must be executed to restore the nominal behavior, e.g., ask the human if it is safe, and is attached to the original AND/OR graph with an AND constraint. Then, the task that has previously failed is reset and marked as a task that must be still scheduled. According to Algorithm 1 in Section 5, with this strategy the scheduler is forced to go through this new restoring path before scheduling again the task that had previously failed.

### 7.2. Non-Restorable Error

When the error compromises the correct execution of the collaborative job, the restoring procedure may not be enough. In this case, it is necessary to ask one of the actors to execute a set of tasks to recover from the failure and continue the collaboration with the next tasks. The procedure implemented in the framework is illustrated in Figure 3c.

A new graph path is inserted that goes from the previous task to the task that follow the failed one. This path is composed by all the tasks necessary to correct from the failure, e.g., adjust the orientation, and it is attached with an OR constraint. According to Algorithm 1 in Section 5, this new path allows the scheduler to continue the collaborative job, omitting the previously failed task.

## 8. Experiments

The proposed framework has been experimentally validated in a human-robot collaborative scenario, where the human operator works together with a Franka Emika Panda, a 7-DoF collaborative robot. Several experiments have been carried out, focusing on four situations of which a video is available as supplementary material:

- Different human operator skills;
- Substitution of human actions with robot action;
- Error handling;
- Parallelism of the actors with resource sharing.

**Figure 4:**
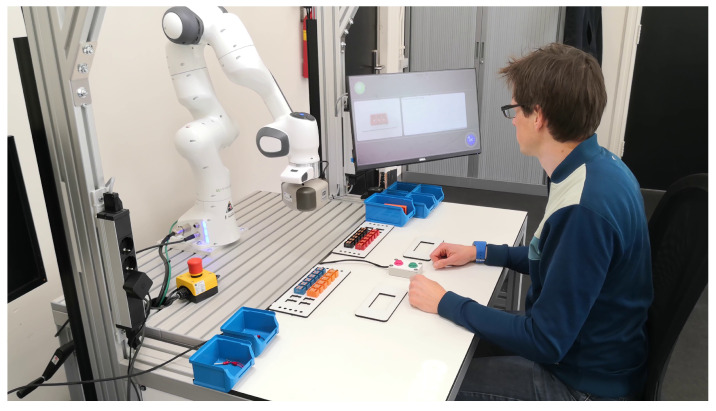
Setup of the experiment. The image shows all the equipment used during the experiments.

During the experiments, the human operator has been guided exploiting the Arkite’s Human Interface Mate (HIM), which also enables the interaction. The complete setup for the experiment is shown in Figure 4.

All the software components were developed in Python 3.8 and exploiting Apache Kafka, while the Franka Emika Panda is position controlled using the MoveIt Motion Planning Framework and the standard ROS libraries.

**Figure 5:**
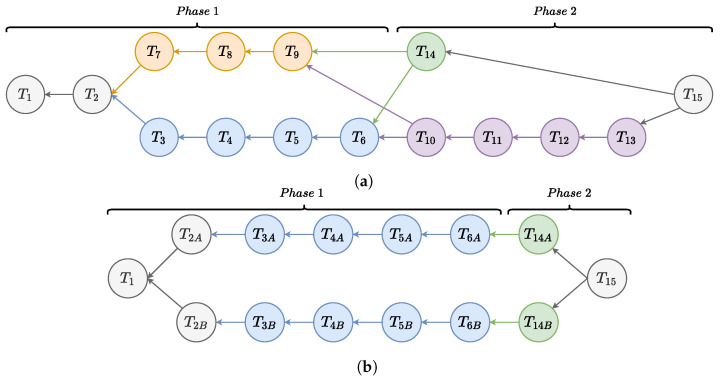
AND/OR graphs representing all the experiments performed. (**a**) Representation of the first three scenarios; (**b**) representation of the parallel work.

The first three situations can be represented with the AND/OR tree shown in Figure 5a, where different paths can be distinguished. The yellow one requires only the human operator to perform the job, while on the blue path, the robot performs some of the tasks. In the last part, the tree divides based on the expertise of the human operator. A detailed description of all the tasks composing the collaborative job is shown in Table 1. It is worth noting that the tasks related to the pick and place of both the casing and the connectors are part of the first phase, while the ones related to the wiring are part of the second phase. The parallel work situation, instead, is schematized in Figure 5b. This represents a double assembly job where both a robot and an expert human operator are available. For ease of reading, the tasks that have been doubled are denoted with the subscripts *A* and *B*.

### 8.1. Different Skills

These experiments mainly focus on the second phase of the collaborative scenario. Initially, the operator is inexperienced in the collaborative work to be pursued. The collaboration starts when the operator confirms being ready and the scheduler immediately asks to place the casing in the correct spot, i.e., $T_{1}$ and $T_{2}$. At this point, the robot starts to pick and place all the connectors, while the human operator is waiting. This is because no parallel tasks are available, see the blue path in Figure 5a. Once the robot has completed the tasks, the collaborative job continues with the second phase. Since the operator is learning, the operator receives detailed step-by-step instructions about the wiring, represented by the purple path in Figure 5b. Exploiting the input coming from the human capabilities block, the scheduler is aware of the human expertise level.

After multiple executions of the collaborative job, the human operator became more expert, and the *Human Capabilities* block detects an upgrade of the user level. In this context, displaying the wiring instructions step by step may be tedious and annoying, with a consequent reduction of the job quality. The scheduler can adapt and chooses the green path in Figure 5a, which merges $T_{10}-T_{13}$ in one single task $T_{14}$ so the operator only receives high level instructions.

This experiment validates the functionality of the checkLevel() constraint presented in Section 6, demonstrating that the overall framework can adapt to different human operators.

### 8.2. Actor Substitution

This experiment simulates the situation where, for whatever reason, the robot is unavailable. This may happen when the tool of the robot is under maintenance, and a fallback strategy is required. In the analyzed scenario, this applies to the robot tasks during the first phase. To simulate such unavailability, the robot actor is removed from the actors list and, without changing the tree, the job is started. As before, the human operator confirms and places the casing in the correct position. At this point, the scheduler can only go through the only human path, the yellow one in Figure 5a, asking the human operator to pick and place the connectors.

### 8.3. Error Handling

In this experiment, the human operator intentionally triggers robot errors, which are subsequently handled by the framework. The framework uses the task monitoring block of Section 7 to detect the correct execution of the task. The first time a task fails, a restorable error is triggered. If the same task fails another time, a non-restoring action is required.

**Figure 6:**
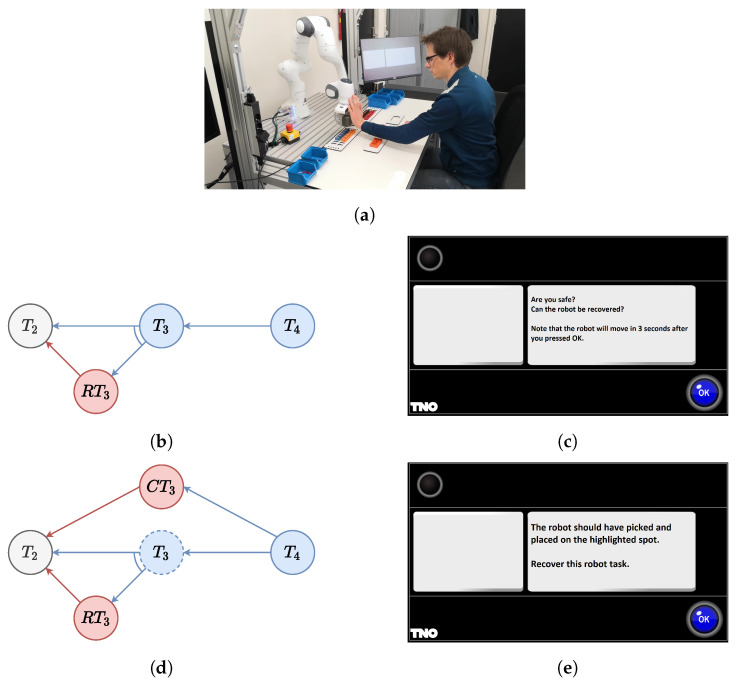
Failure of of $T_{3}$. (**a**) The human hindering the robot. (**b**,**d**) The AND/OR graph locally adapted. In (**c**), the Arkite system asks if the human is safe before moving again, while in (**e**), the instruction to guide the human operator in correcting the task is shown.

During the execution of $T_{3}$, the human operator hinders the robot, which immediately stops for safety reasons. At this point, the tree is locally adapted by inserting a restoring task. Thanks to this task, the scheduler firstly asks the human operator if they are safe and, if possible, moves the robot to the home position ready to retry the execution. This is shown in Figure 6b,c. Subsequently, the human operator hinders the robot again, causing another failure. Since the task failed twice, the task monitoring generates a not restorable error. This is because it would be better to ask the human operator to execute this task. For this reason, the AND/OR graph is modified again adding a new path to reach $T_{4}$. As before, the human operator must confirm that everything is fine and the robot goes back in a home position, but this time the scheduler asks the human to pick and place the connector in the correct place. All the steps are illustrated in Figure 6d,e. From this point, the robot can resume its work.

### 8.4. Parallel Work

The previous situations validate the effectiveness of the framework and its features in a sequential scenario. The framework is also capable of scheduling parallel tasks. In this case, the robot works in parallel, sharing resources with the human operator. This is shown in the last scenario, where the human and the robot work on two products at the same time while sharing a workspace. Without losing generality, the scenario has been simplified avoiding the possibility of different paths.

When the collaborative job starts, the scheduler immediately asks the human operator to pick and place the first casing. Subsequently, the robot starts to insert the three connectors while the human operator places the second casing. Once the robot has finished working on the first casing, the scheduler asks the human to make the wiring. It is worth noting that since the human operator is an expert user, the wiring is composed by a single task. In the meantime, the robot finishes putting the connectors inside the second casing, allowing the human to conclude the job with the second wiring.

## 9. Conclusions and Future Works

In this paper, a framework for resilient and effective task scheduling in a collaborative industrial scenario has been built. With this framework, industrial collaborative human-robot processes can be modeled and executed. This includes the nominal execution behavior as well as alternative execution behaviors that might be caused by common situations at the workplace. These situations include adjusting to the skill level of the operator, actor substitution, and error handling.

Starting from the definition of the tasks composing the collaborative job, namely the AND/OR graph, the scheduler is responsible for deciding what is the most suitable task for the actors to execute at each point in time. The task monitoring strategy is exploited to understand if the task has been correctly executed and, if necessary, to locally adapt the AND/OR graph to prevent that a task failure will result in a halted process. Moreover, the scheduler exploits the information about the human expertise to improve the HRC. In the paper, the methodologies used to online estimate the human expertise have not been investigated. In this paper we have used a description that included a single human operator and robot, but the framework can be extended to larger teams without major modifications.

The experimental validation has been conducted in a scenario with different execution variations, showing and proving that the proposed framework handles most situations that may occur in a real industrial setting.

Future works aim at improving the scheduling strategy in order to also choose the path that optimizes a desired cost function, e.g., exploiting the A* algorithm over the AND/OR graph [12]. Moreover, the framework should be tested in a user study that involves a real-world industrial scenario. This would lead to a further validation and improvement of the overall framework. Lastly, the framework could be extended to also handle the synchronization between the actors. In this scenario, some strategies such as [38,39] could be used to enable the mutual communication between the two actors.

## Figures and Tables

**Table 1:** Tasks description of AND/OR graph in Figure 5a.

Task Index	Description	Agent	Phase
1	Start the job.	*H*	Phase 1
2	Pick and Place casing.	*H*	
3	Move in Home.	*R*	
4–6	Robot Pick and Place 3 connectors.	*R*	
7–9	Human Pick and Place 3 connectors.	*H*	
10–13	Connect 4 wires one by one—not expert user.	*H*	Phase 2
14	Connect all the wires—expert user.	*H*	
15	Confirm completion.	*H*	

## Data Availability

Not applicable.

## Supplementary Materials

The following supporting information can be downloaded at: https://www.mdpi.com/article/10.3390/s22134901/s1, Video S1: A Resilient and Effective Task Scheduling Approach for Industrial Human-Robot Collaboration.

## References

[1] Villani V., Pini F., Leali F., Secchi C. (2018). Survey on human—Robot collaboration in industrial settings: Safety, intuitive interfaces and applications. Mechatronics.

[2] Pham Q., Madhavan R., Righetti L., Smart W., Chatila R. (2018). The impact of robotics and automation on working conditions and employment. IEEE Robot. Autom. Mag..

[3] Pupa A., Arrfou M., Andreoni G., Secchi C. (2021). A Safety-Aware Kinodynamic Architecture for Human-Robot Collaboration. IEEE Robot. Autom. Lett..

[4] Benzi F., Secchi C. An Optimization Approach for a Robust and Flexible Control in Collaborative Applications. Proceedings of the 2021 IEEE International Conference on Robotics and Automation (ICRA).

[5] Merckaert K., Convens B., Wu C.J., Roncone A., Nicotra M.M., Vanderborght B. (2022). Real-time motion control of robotic manipulators for safe human—Robot coexistence. Robot. Comput.-Integr. Manuf..

[6] Pupa A., Landi C.T., Bertolani M., Secchi C., Saveriano M., Renaudo E., Rodríguez-Sánchez A., Piater J. (2021). A Dynamic Architecture for Task Assignment and Scheduling for Collaborative Robotic Cells. Human-Friendly Robotics 2020.

[7] Sheridan T.B. (2016). Human-Robot Interaction: Status and Challenges. Hum. Factors J. Hum. Factors Ergon. Soc..

[8] Hoffman G. (2019). Evaluating Fluency in Human-Robot Collaboration. IEEE Trans. Hum.-Mach. Syst..

[9] He Y., Shao Z., Xiao B., Zhuge Q., Sha E. Reliability driven task scheduling for heterogeneous systems. Proceedings of the Fifteenth IASTED International Conference on Parallel and Distributed Computing and Systems.

[10] Qin X., Xie T. (2008). An availability-aware task scheduling strategy for heterogeneous systems. IEEE Trans. Comput..

[11] Makrini I.E., Merckaert K., Winter J.D., Lefeber D., Vanderborght B. (2019). Task allocation for improved ergonomics in Human-Robot Collaborative Assembly. Interact. Stud..

[12] Pratissoli F., Battilani N., Fantuzzi C., Sabattini L. Hierarchical and Flexible Traffic Management of Multi-AGV Systems Applied to Industrial Environments. Proceedings of the 2021 IEEE International Conference on Robotics and Automation (ICRA).

[13] Michalos G., Spiliotopoulos J., Makris S., Chryssolouris G. (2018). A method for planning human robot shared tasks. CIRP J. Manuf. Sci. Technol..

[14] Li K., Liu Q., Xu W., Liu J., Zhou Z., Feng H. (2019). Sequence Planning Considering Human Fatigue for Human-Robot Collaboration in Disassembly. Procedia CIRP.

[15] Ayough A., Zandieh M., Farhadi F. (2020). Balancing, Sequencing, and Job Rotation Scheduling of a U-Shaped Lean Cell with Dynamic Operator Performance. Comput. Ind. Eng..

[16] Klein G., Woods D.D., Bradshaw J.M., Hoffman R.R., Feltovich P.J. (2004). Ten challenges for making automation a “team player” in joint human-agent activity. IEEE Intell. Syst..

[17] Johnson M., Bradshaw J.M., Feltovich P.J., Jonker C.M., Van Riemsdijk M.B., Sierhuis M. (2014). Coactive Design: Designing Support for Interdependence in Joint Activity. J. Hum.-Robot Interact..

[18] Ramamritham K. (1995). Allocation and scheduling of precedence-related periodic tasks. IEEE Trans. Parallel Distrib. Syst..

[19] Gacias B., Artigues C., Lopez P. (2010). Parallel machine scheduling with precedence constraints and setup times. Comput. Oper. Res..

[20] Zhang K., Qi B., Jiang Q., Tang L. Real-time periodic task scheduling considering load-balance in multiprocessor environment. Proceedings of the 2012 3rd IEEE International Conference on Network Infrastructure and Digital Content.

[21] Yu T., Huang J., Chang Q. (2021). Optimizing task scheduling in human-robot collaboration with deep multi-agent reinforcement learning. J. Manuf. Syst..

[22] Ferreira C., Figueira G., Amorim P. (2021). Scheduling Human-Robot Teams in collaborative working cells. Int. J. Prod. Econ..

[23] Nourmohammadi A., Fathi M., Ng A.H. (2022). Balancing and scheduling assembly lines with human-robot collaboration tasks. Comput. Oper. Res..

[24] Rahman S.M., Wang Y. (2018). Mutual trust-based subtask allocation for human–robot collaboration in flexible lightweight assembly in manufacturing. Mechatronics.

[25] Nikolakis N., Kousi N., Michalos G., Makris S. (2018). Dynamic scheduling of shared human-robot manufacturing operations. Procedia CIRP.

[26] Pupa A., Van Dijk W., Secchi C. (2021). A Human-Centered Dynamic Scheduling Architecture for Collaborative Application. IEEE Robot. Autom. Lett..

[27] Pupa A., Secchi C. A Safety-Aware Architecture for Task Scheduling and Execution for Human-Robot Collaboration. Proceedings of the 2021 IEEE/RSJ International Conference on Intelligent Robots and Systems (IROS).

[28] Raatz A., Blankemeyer S., Recker T., Pischke D., Nyhuis P. (2020). Task scheduling method for HRC workplaces based on capabilities and execution time assumptions for robots. CIRP Ann..

[29] Johannsmeier L., Haddadin S. (2016). A hierarchical human-robot interaction-planning framework for task allocation in collaborative industrial assembly processes. IEEE Robot. Autom. Lett..

[30] Zhang M., Li C., Shang Y., Liu Z. (2022). Cycle Time and Human Fatigue Minimization for Human-Robot Collaborative Assembly Cell. IEEE Robot. Autom. Lett..

[31] De Mello L.H., Sanderson A.C. A correct and complete algorithm for the generation of mechanical assembly sequences. Proceedings of the 1989 IEEE International Conference on Robotics and Automation. IEEE Computer Society.

[32] Homem de Mello L., Sanderson A. (1990). AND/OR graph representation of assembly plans. IEEE Trans. Robot. Autom..

[33] Canham R., Jackson A., Tyrrell A. Robot error detection using an artificial immune system. Proceedings of the NASA/DoD Conference on Evolvable Hardware.

[34] Pettersson O. (2005). Execution monitoring in robotics: A survey. Robot. Auton. Syst..

[35] Trung P., Giuliani M., Miksch M., Stollnberger G., Stadler S., Mirnig N., Tscheligi M. Head and shoulders: Automatic error detection in human-robot interaction. Proceedings of the 19th ACM International Conference on Multimodal Interaction.

[36] Vo N.N., Bobick A.F. From stochastic grammar to bayes network: Probabilistic parsing of complex activity. Proceedings of the IEEE Conference on Computer Vision and Pattern Recognition.

[37] Maeda G., Ewerton M., Neumann G., Lioutikov R., Peters J. (2017). Phase estimation for fast action recognition and trajectory generation in human—Robot collaboration. Int. J. Robot. Res..

[38] Muthugala M., Srimal P., Jayasekara A. (2021). Improving robot’s perception of uncertain spatial descriptors in navigational instructions by evaluating influential gesture notions. J. Multimodal User Interfaces.

[39] Capelli B., Villani V., Secchi C., Sabattini L. Understanding multi-robot systems: On the concept of legibility. Proceedings of the 2019 IEEE/RSJ International Conference on Intelligent Robots and Systems (IROS).

